# Heart rate variability in type 2 diabetes mellitus: A systematic review and meta–analysis

**DOI:** 10.1371/journal.pone.0195166

**Published:** 2018-04-02

**Authors:** Thomas Benichou, Bruno Pereira, Martial Mermillod, Igor Tauveron, Daniela Pfabigan, Salwan Maqdasy, Frédéric Dutheil

**Affiliations:** 1 University Hospital of Clermont–Ferrand, CHU Clermont–Ferrand, Endocrinology, Clermont–Ferrand, France; 2 University Hospital of Clermont–Ferrand, CHU Clermont–Ferrand, Clinical Research Direction, Clermont–Ferrand, France; 3 Univ. Grenoble Alpes, LPNC & CNRS, LPNC, Grenoble, France; 4 Institut Universitaire de France, Paris, France; 5 Université Clermont Auvergne, CNRS, GReD, Inserm, University Hospital of Clermont–Ferrand, CHU Clermont–Ferrand, Endocrinology, Clermont–Ferrand, France; 6 Peking University, Culture and Social Cognitive Neuroscience Laboratory, School of Psychological and Cognitive Sciences, Beijing, China; 7 Université Clermont Auvergne, CNRS, LaPSCo, Physiological and Psychosocial Stress, University Hospital of Clermont–Ferrand, CHU Clermont–Ferrand, Preventive and Occupational Medicine, WittyFit, Clermont–Ferrand, France; 8 Australian Catholic University, Faculty of Health, School of Exercise Science, Melbourne, Victoria, Australia; Weill Cornell Medicine-Qatar, QATAR

## Abstract

**Background:**

Cardiac autonomic neuropathy in type 2 dibetes mellitus (T2DM) patients is frequent and associated with high cardiovascular mortality. Heart rate variability (HRV) is the gold standard to measure cardiac autonomic neuropathy. We aimed to conduct a systematic review and meta–analysis to evaluate the impact of T2DM on HRV parameters.

**Methods:**

The PubMed, Cochrane Library, Embase and Science Direct databases were searched on 1^st^ October 2017 using the keywords “diabetes” AND (“heart rate variability” OR “HRV”). Included articles had to report HRV parameters in T2DM patients and healthy controls measured during 24 hours with a Holter–electrocardiogram. Measurements of HRV retieved were: RR–intervals (or Normal to Normal intervals—NN), standard deviation of RR intervals (SDNN), percetange of adjacent NN intervals differing by more than 50 milliseconds (pNN50), square root of the mean squared difference of successive RR intervals (RMSSD), total power, Low Frequency (LF), High Frequency (HF) and LF/HF ratio, as per Task Force recommendations.

**Results:**

We included twenty-five case-control studies with 2,932 patients: 1,356 with T2DM and 1,576 healthy controls. T2DM patients had significantly (P<0.01) lower RR–intervals (effect size = –0.61; 95%CI –1.21 to –0.01), lower SDNN (–0.65; –0.83 to –0.47), lower RMSSD (–0.92; –1.37 to –0.47), lower pNN50 (–0.46; –0.84 to –0.09), lower total power (–1.52; –2.13 to –0.91), lower LF (–1.08; –1.46 to –0.69]), and lower HF (–0.79; –1.09 to –0.50). LF/HF did not differ between groups. Levels of blood glucose and HbA1c were associated with several HRV parameters, as well as Time from diagnosis of T2DM

**Conclusions:**

T2DM was associated with an overall decrease in the HRV of T2DM patients. Both sympathetic and parasympathetic activity were decreased, which can be explained by the deleterious effects of altered glucose metabolism on HRV, leading to cardiac autonomic neuropathy.

## Introduction

Type 2 diabetes mellitus (T2DM) is a public health concern [1]. T2DM is increasingly frequent in the world in association with the increase of sedentary behaviours, unhealthy diet, obesity and metabolic syndrome [2–6]. The number of people with T2DM is predicted to double within the next three decades [1]. Besides macrovascular [7–10] and microvascular complications [11–13], the leading cause of death in T2DM is cardiovascular mortality [9]. Cardiovascular mortality has been related to the cardiac autonomic neuropathy frequently associated with T2DM [1,5,14].

Screening for cardiac autonomic neuropathy has been recommended at the diagnosis of T2DM, particularly in patients with a history of poor glycaemic control, macro/micro vascular complications, and increased cardiovascular risk [15]. Despite standard cardiovascular reflex tests still belong to the gold standard for the assessment of cardiovascular autonomic neuropathy [16], one of the easiest and most reliable ways to assess cardiac autonomic neuropathy is through the measurement of heart rate variability (HRV). HRV is the variation between two consecutive beats: the higher the variation, the higher the parasympathetic activity. A high HRV reflects the fact that an individual can constantly adapt to micro–environmental changes [17]. Therefore, low HRV is a marker of cardiovascular risk [18]. Conveniently, the measurement of HRV is non–intrusive and pain–free [19]. Although the evaluation of HRV in T2DM has been assessed in several studies, conflicting results have been reported [20–22]. Moreover, there is no consensus on the decreased levels of HRV parameters in T2DM. Furthermore, despite HRV being linked with the severity of T2DM [23], no studies have comprehensively assessed the role of the most common variables, such as age [24], gender [25], blood glucose control [26], or medications treating for T2DM, on HRV parameters [27,28].

Therefore, we aimed to conduct a systematic review and meta–analysis on the impact of T2DM on HRV parameters. A secondary aim was to identify the most frequently reported explanatory variables.

## Methods

### Literature search

**Fig 1:**
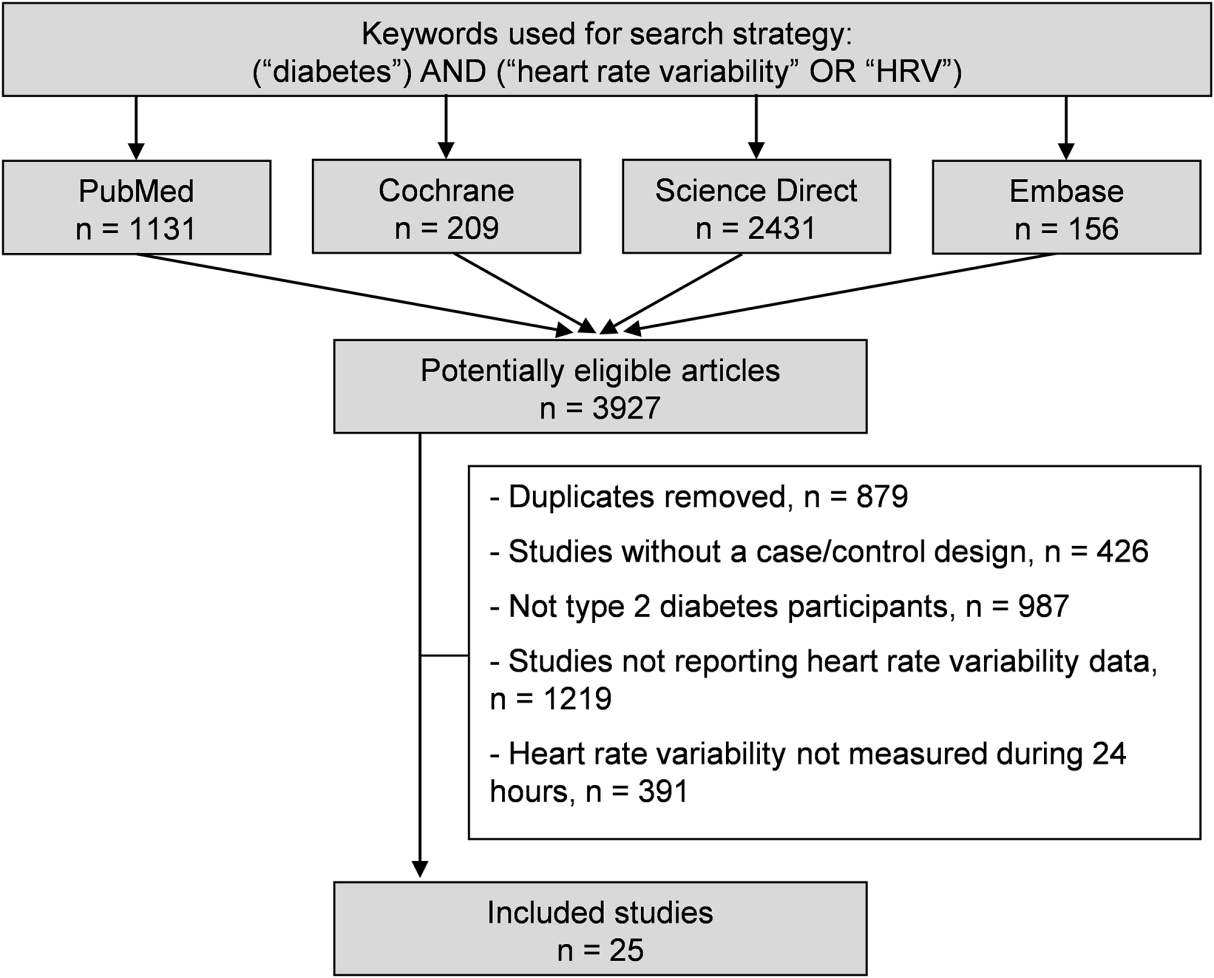
Search strategy.

We reviewed all studies measuring HRV in T2DM patients and healthy controls. Animal studies were excluded. Between October 30^th^ 2015 and October 1^st^ 2017, the main articles databases (PubMed, Cochrane Library, Science Direct and Embase) were searched with the following keywords: “diabetes” AND (“heart rate variability” OR “HRV”). All articles compatible with our inclusions criteria were included, independently of article language and years of publication. To be included, case-control studies had to describe our main primary outcome, which was the measurement of HRV parameters in T2DM patients and healthy controls. We limited included studies to those reporting 24–hour measurements of HRV with Holter–electrocardiogram, following Task Force recommendations [29]. We imposed no limitation on the regional origin or the nature of the control group. Studies needed to be primary research. In addition, reference lists of all publications meeting the inclusion criteria were manually searched to identify any further studies that were not found with the electronic search. Ancestry searches were also completed on previous reviews to locate other potentially eligible primary studies. The search strategy is presented in Fig 1 and in S1 Appendix. One author (Thomas Benichou) conducted the literature searches, collated the articles, and extracted the data. Two authors (Thomas Benichou and Frédéric Dutheil) reviewed the abstracts independently and checked if article could be included in our metanalaysis according to inclusion critera. When consensus on suitability was not reached, a third author (Bruno Pereira) reviewed the debated articles. Then, all authors reviewed the eligible articles.

### Quality of the assessment

Despite not designed for quantifying the integrity of studies [30], the “STrengthening the Reporting of OBservational studies in Epidemiology” (STROBE) was used for checking the reporting quality of cohorts studies [31]. The STROBE Statement is a checklist of 22 items related to the title, abstract, introduction, methods, results and discussion sections of articles. Cohort, case control, and cross-sectional studies shared 18 items. Four items are specific to each of the three study designs. Six of the 22 items are splitted into several sub-items. We attributed one point per item or sub-item fulfilling the criteria. We calculated a percentage on a maximal score achievable of 32. The Consolidated Standards of Reporting Trials (CONSORT) for checking the reporting quality of randomized trials [32]. Similarly, the 25 items (with 11 of them splitted into several sub-items) from the CONSORT criteria could achieve a maximum score of 37, then converted into percentage.

### HRV parameters

We included HRV parameters derived from a 24–hour Holter electrocardiogram following task force recommendations [29]. In the time domain, we analysed RR intervals, standard deviations of RR intervals (SDNN), the square root of the mean squared difference of successive RR intervals (RMSSD), and the percentage of adjacent NN intervals differing by more than 50 milliseconds (pNN50). The RMSSD and pNN50 are associated with high–frequency power (HF) and hence parasympathetic activity, whereas SDNN is correlated with low–frequency power (LF) [33]. In the spectral domain, we analysed LF (0.04–0.15 Hz), an index of both sympathetic and parasympathetic activity, and HF (0.15–0.4 Hz), representing the most efferent vagal (parasympathetic) activity to the sinus node. Very low frequency (VLF; 0.003–0.04 Hz) partially reflects thermoregulatory mechanisms, fluctuation in activity of the renin–angiotensin system, and the function of peripheral chemoreceptors. The LF/HF ratio, i.e. the sympathovagal balance, was also calculated.

### Statistical considerations

We conducted meta–analyses on the HRV parameters in T2DM patients and healthy controls. P values less than 0.05 were considered statistically significant. For the statistical analysis, we used both Comprehensive Meta–analysis software (version 2, Biostat Corporation) [34–37] and Stata software (version 13, StataCorp, College Station, US) [34–36,38,39]. Main characteristics were synthetized for each study population and reported as the mean ± SD (standard–deviation) for continuous variables and the number (%) for categorical variables.

We evaluated heterogeneity in the study results by examining forest plots, confidence intervals (CI) and I^2^ statistic. Formal tests for homogeneity based on the I^2^ statistic are the most common metric for measuring the magnitude of between–study heterogeneity and are easily interpretable. I^2^ values range from 0 to 100%, and are considered low for <25%, modest for 25–50%, and high for >50%. We assume heterogeneity for a p–value of the I^2^ test <0.05. For example, a significant heterogeneity could be linked to the characteristics of the studies, such as those of the participants (age, sex, etc.), the time from T2DM diagnosis, the glycaemia or the HbA1c levels. We conducted random effects meta–analyses (DerSimonian and Laird approach) when data could be pooled [40]. To describe our results, we calculated the effect size (ES, standardised mean differences—SMD) [41] of each HRV parameter for each dependent variable. An ES is a unitless measure of the levels of the HRV data. The ES is centered at zero if the HRV data in T2DM patients are not different from those in healthy controls. A positive ES denoted higher levels of the tested HRV parameter in T2DM patients compared with heathy controls. An ES of 0.8 reflects a large effect, 0.5 a moderate effect, and 0.2 a small effect. We searched for potential publication bias using funnel plots of these meta–analyses. We verified the strength of our results by conducting further meta–analyses after exclusion of studies that were not evenly distributed around the base of the funnel.

When possible (sufficient sample size), meta–regressions were proposed to study the relationship between each HRV parameter (RR intervals, RMSSD, pNN50, SDNN, total power, LF, HF, LF/HF) and clinically relevant parameters such as gender, age, fasting blood glucose, and glycated haemoglobin (HbA1c). Results were expressed as regression coefficients and 95% CI.

## Results

An initial search produced a possible 3927 articles (Fig 1). Removal of duplicates and use of the selection criteria reduced the number of articles reporting the evaluation of HRV on 24-hour recording in T2DM to 25 articles [42–66]. All included articles were written in English except one study which was written in Hungarian [55].

### Quality of articles

The assessment of the quality of the twenty–five studies that were included was performed using the STROBE and CONSORT criteria. Results varied from 50% [59] to 71% [56] for the observational studies (STROBE), with a mean score of 59%. Results varied from 52% [59] to 67% [57] for the randomized trials (CONSORT), with a mean score of 59.5%. Overall, the studies performed best in the methods section and worst in the discussion section.

### Objectives of included articles

All included articles aimed to compare HRV between T2DM patients and controls without T2DM [42–66]. Both the T2DM patients and the controls had cardiovascular diseases in five studies [42,48,51,56,60] and renal disease in three studies [43,46,52]. Five studies assessed the influence of high blood pressure on HRV in T2DM compared with healthy controls [49,50,54,55,58]. Other studies compared HRV between T2DM patients and controls based on blood catecholamine levels [44], circadian autonomic rhythm in insulinoresistant subjects [45], in cases of bowel preparation [63], metabolic syndrome [47], circadian rhythm in relation to blood adiponectin [62], or dimethylarginine levels [61], hypoglycaemic episodes [59], in acromegalic patients [53], and inhalation of ultrafine particles [57].

### Inclusions and exclusions criteria

*For T2DM patients*: Inclusion criteria were: aged over 18 [43,48], and under 65 [51], 73 [53], or 75 years old [46,52], treated with oral antidiabetic agents [47], with normal [42,44,45] or high blood pressure [49,61]. The main exclusion criteria were: pregnancy [43,62], neurological disease [43]. Body Mass Index (BMI) over 25 kg/m^2^ [42,44,45], or 35 kg/m^2^ [58,62], chronic heart [42,44,45,50–52,57,58,60–63], liver [56,60,63], or renal [42,42,45,56,58,60–63] failure, insulin treatment [47,51], uncontrolled T2DM [47], thyroid disorder [50,52], or treatment that can influence HRV parameters [44–46,52,54,57–60].

*For controls*: All studies included controls without T2DM [42–66]. In each individual study, exclusion criteria were the same as for T2DM, i.e. pregnancy [43,62], neurological disease [43], BMI over 25 kg/m^2^ [42,44,45] or 35 kg/m^2^ [58,62], chronic heart [42,44,45,50–52,57,58,60–63], liver [56,60,63], or renal [42,42,45,56,58,60–63] failure, thyroid disorder [50,52], or treatment that can influence HRV parameters [44–46,52,54,57–60]. Controls had high blood pressure in five studies [49,56,58,60,62], and were on dialysis in three studies [43,46,52].

Healthy controls were paired with T2DM patients based on age [41,43,44,46,47,49,50,52,53,58,59,62], gender [42,44,45,47,51,59,60,63], body weight [50,59], BMI [44], and blood pressure [60].

### Population

#### Sample size

Population sizes ranged from 12 [59], to 457 [56]. We included 2,932 patients in total: 1,356 with T2DM and 1,576 healthy controls.

#### Gender

The proportion of men varied from 35% [45] to 100% [50] in T2DM patients, with a mean of 52.2%, and from 28% [54] to 100% [60] in the control group, with a mean of 54.5%. One study did not specify the proportion of men with T2DM [53], while 3 studies did not specify it for the controls [53,57,59].

#### Age

The mean age of T2DM patients was 58.1±6.5 years, ranging from 45.9 [57] to 67.9 [52], and 55.9±7.6 years in the controls, ranging from 28.5 [57] to 65.7 [52]. Age was not reported for T2DM patients in two studies [50,53], and for the controls in three studies [50,53,59].

*T2DM duration*: the mean time from T2DM diagnosis was 7.8±4.4 years ranging from 3.0 [47,54] to 11.2 [51] years. T2DM duration was not reported in five studies [45,48,50,52,53].

#### Metabolic control

The mean *HbA1c* in T2DM patients was 7.6±0.8%, ranging from 6.5 [59] to 9.3% [58,62], and 5.2±0.5% in the controls, ranging from 4.1 [42] to 5.8% [58,62]. Eight studies did not report HbA1c in the T2DM patients [43,45,46,48,50,53,61,64] and nine studies did not specify for the controls [43,45–48,50,53,61,64]. *Blood glucose levels* were reported in 14 studies [42,44,46,47,51,52,55,58,60–63,65,66] with a mean blood glucose level of 147±16 mg/dL in T2DM patients, ranging from 117 [47] to 168 [58,62], and 90±6 in controls, ranging from 75 [46] to 95 [55]. Blood insulin levels were reported in one study [44]. No studies reported HOMA–IR. No studies reported neuropathy scores.

#### Body weight

Weight was reported in three studies [43,46,49], and waist circumference in two studies [47,52]. The mean BMI in T2DM patients was 27.2±3.5 kg/m^2^, ranging from 22.3 [44] to 33.7 [58,62], and 24.8±2.1 in controls, ranging from 21.8 [61] to 29.7 [54]. BMI was not reported in 11 studies in T2DM patients [42,43,46,48,50–53,55,57,64], and in 12 studies in controls [42,43,46,48,50–53,55,57,59,64].

#### Blood pressure

Mean systolic blood pressure in T2DM patients was 138.6±11.0 mmHg, ranging from 121.2 [44] to 156.0 [49], and 137.7±11.6 mmHg in controls, ranging from 120.2 [45] to 153.0 [49]. Mean diastolic blood pressure in T2DM patients was 80.8±4.3 mmHg, ranging from 69.9 [50] to 90.1 [49] and 82.2±5.4 mmHg in controls, ranging from 71.9 [50] to 92.9 [58,62]. Six studies did not report blood pressure in either T2DM patients or controls [42,48,52,53,57,64].

#### Blood lipid levels

Total cholesterol was reported in nine studies [42,47,51,54,55,61,62,65,66]. HDL and LDL cholesterol in six studies [47,55,60–62,65] and triglycerides in 12 studies [42,47,51,55,56,58–62,65,66].

#### Other characteristics

*Smoking* was reported in six studies [44,54–57,60], and *alcohol* in one study [44]. *Marital status* was never reported. Insufficient data precluded further analyses of those parameters.

### Study designs

All studies described a prospective cohort design, except one study which was a randomly controlled prospective study [57].

### HRV measurements and analysis

All included studies measured HRV over 24 consecutive hours using a Holter–electrocardiogram. HRV measurement recording was ambulatory with normal daily activity in most of the studies [42–45,47–51,53,54,56–58,60,62,64–66], ambulatory in non–dialysed patients and during hospitalization for dialysed patients in two studies [46,52], and exclusively during hospitalization in one study [59]. Three studies did not report the conditions of the measurements [55,61,63].

Nearly all studies had a distinct Holter monitoring system: Del Mar Reynolds Medical [61], CardioSmart Institutional CS 550 software [58], Holter cardio Light digital [62], Zymed Medical Instruments [48], Mortara Instruments [53,57], TM 2421 & 2425 systems [56], Holter AD35 TOP [46], Marquette Electronics [43], Meditech Cardiotens [54,55], Fukuda System [44], Spiderview Holter [52], Cardioscan DMS300–4 Model [63], Holter Digital Recorder AsPEKT [42,51], ArguSys Holter Monitor [47], CardioDay GETEMED [59]. Schiller Microvit MT–101 [65,66], and A&D System [49]. Four studies did not report the monitoring system [45,50,60,64], Fifteen studies explicitly mentioned that they followed task force recommandations [42,44,45,47,48,51–56,58,59,61,62]. Premature atrial and ventricular beats were automatically discarded and visually checked.

### Meta–analyses of HRV values in T2DM

**Fig 2:**
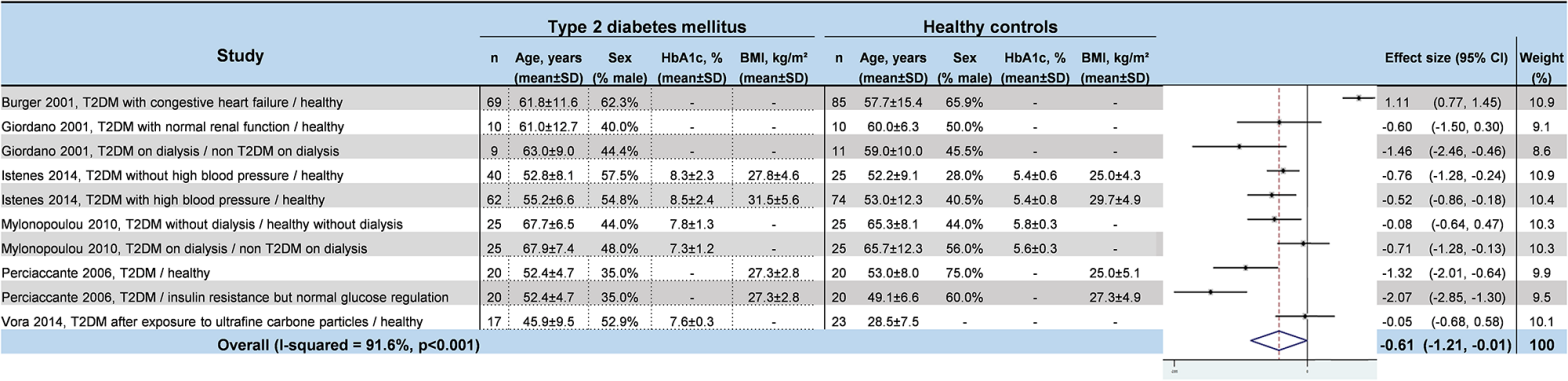
Meta–analysis of RR intervals of type 2 diabetes mellitus patients compared with controls. -: non reported data (missing SD were also non reported). 95% CI: 95% confident intervals; BMI: Body Mass Index; RR: RR intervals; T2DM: Type 2 Diabetes Mellitus; SD: Standard Deviation.

**Fig 3:**
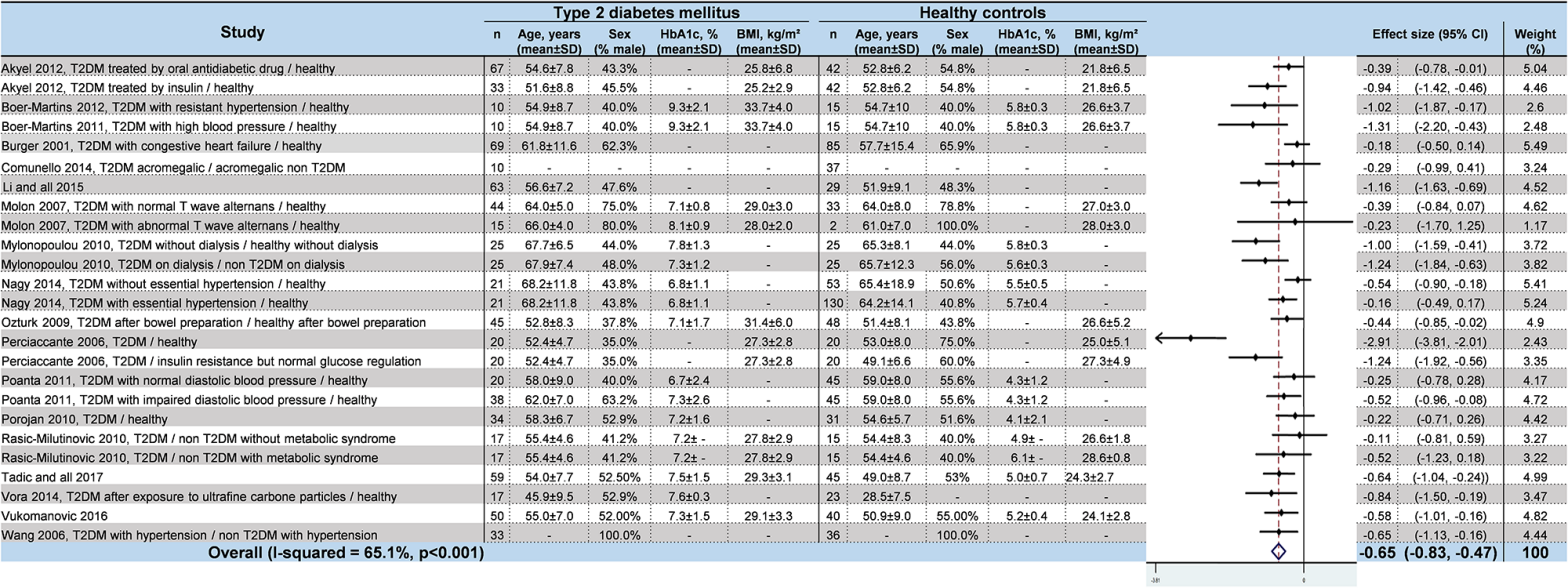
Meta–analysis of SDNN of type 2 diabetes mellitus patients compared with controls. -: non reported data (missing SD were also non reported). 95% CI: 95% confident intervals; BMI: Body Mass Index; SDNN: Standard Deviation of RR intervals; T2DM: Type 2 Diabetes Mellitus; SD: Standard Deviation.

**Fig 4:**
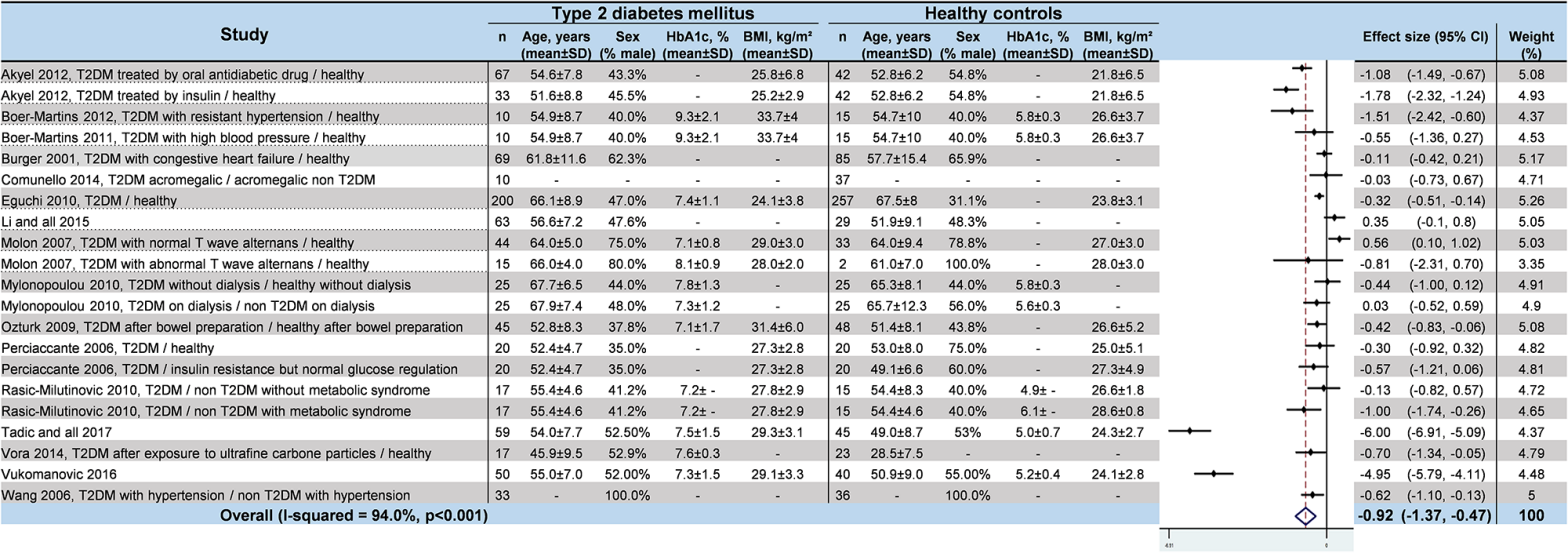
Meta–analysis of RMSSD of type 2 diabetes mellitus patients compared with controls. -: non reported data (missing SD were also non reported). 95% CI: 95% confident intervals; BMI: Body Mass Index; RMSSD: square root of mean squared differences of successive RR intervals; T2DM: Type 2 Diabetes Mellitus; SD: Standard Deviation.

**Fig 5:**
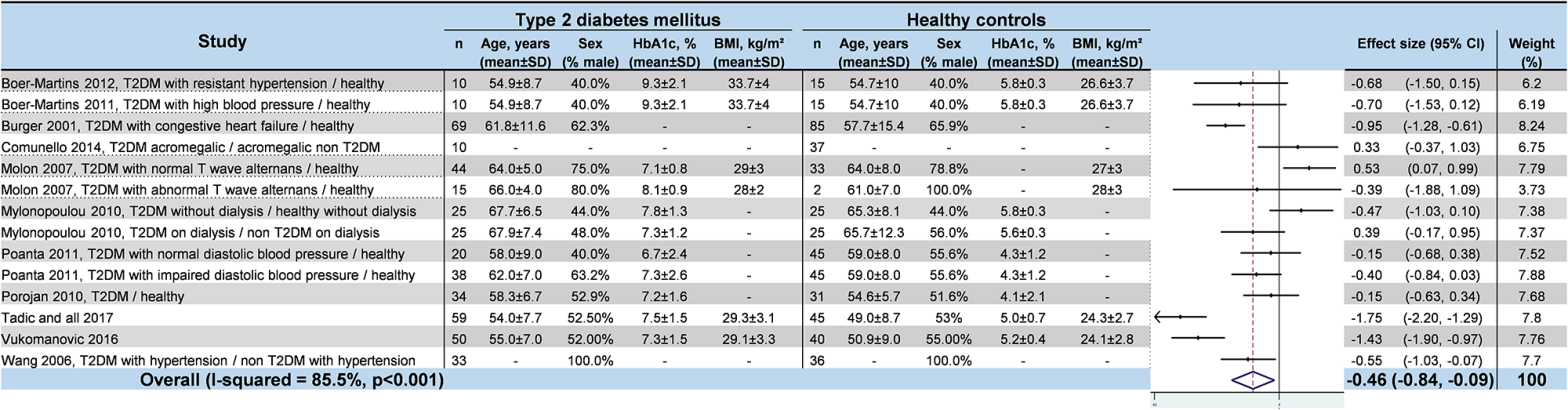
Meta–analysis of pNN50 of type 2 diabetes mellitus patients compared with controls. -: non reported data (missing SD were also non reported). 95% CI: 95% confident intervals; BMI: Body Mass Index; pNN50: percentage of RR intervals with more than 50 ms variation; T2DM: Type 2 Diabetes Mellitus; SD: Standard Deviation.

**Fig 6:**
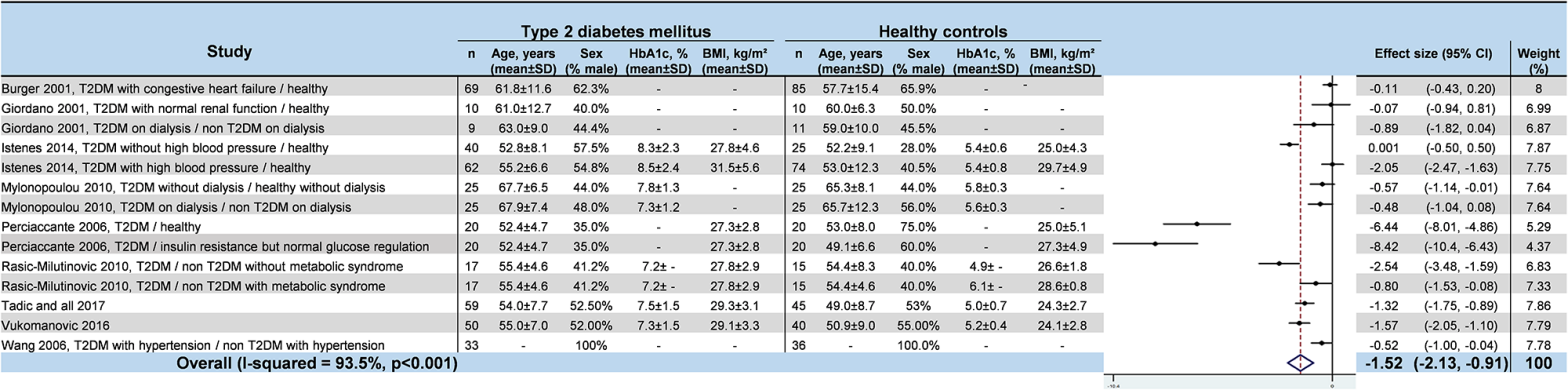
Meta–analysis of total power of type 2 diabetes mellitus patients compared with controls. -: non reported data (missing SD were also non reported). 95% CI: 95% confident intervals; BMI: Body Mass Index; TP: Total Power; T2DM: Type 2 Diabetes Mellitus; SD: Standard Deviation.

**Fig 7:**
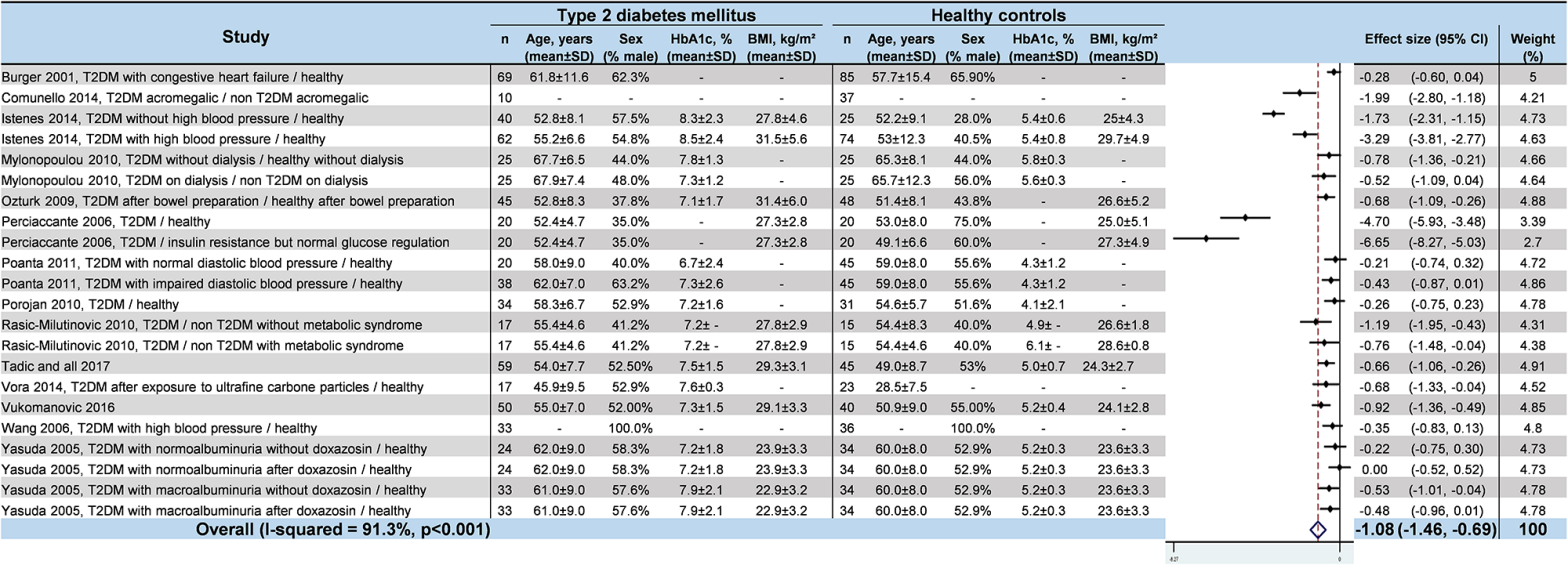
Meta–analysis of the LF of type 2 diabetes mellitus patients compared with controls. -: non reported data (missing SD were also non reported). 95% CI: 95% confident intervals; BMI: Body Mass Index; LF: Low Frequency; T2DM: Type 2 Diabetes Mellitus; SD: Standard Deviation.

**Fig 8:**
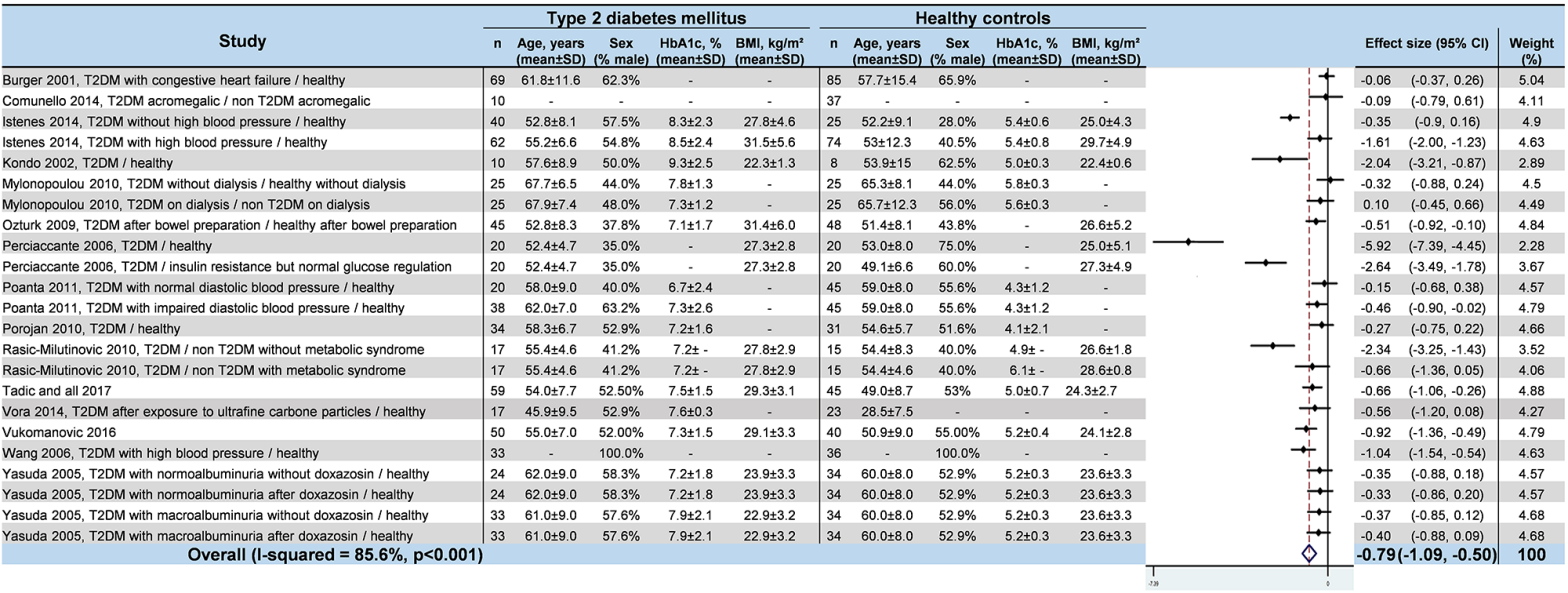
Meta–analysis of the HF of type 2 diabetes mellitus patients compared with controls. -: non reported data (missing SD were also non reported). 95% CI: 95% confident intervals; BMI: Body Mass Index; HF: High Frequency; T2DM: Type 2 Diabetes Mellitus; SD: Standard Deviation.

**Fig 9:**
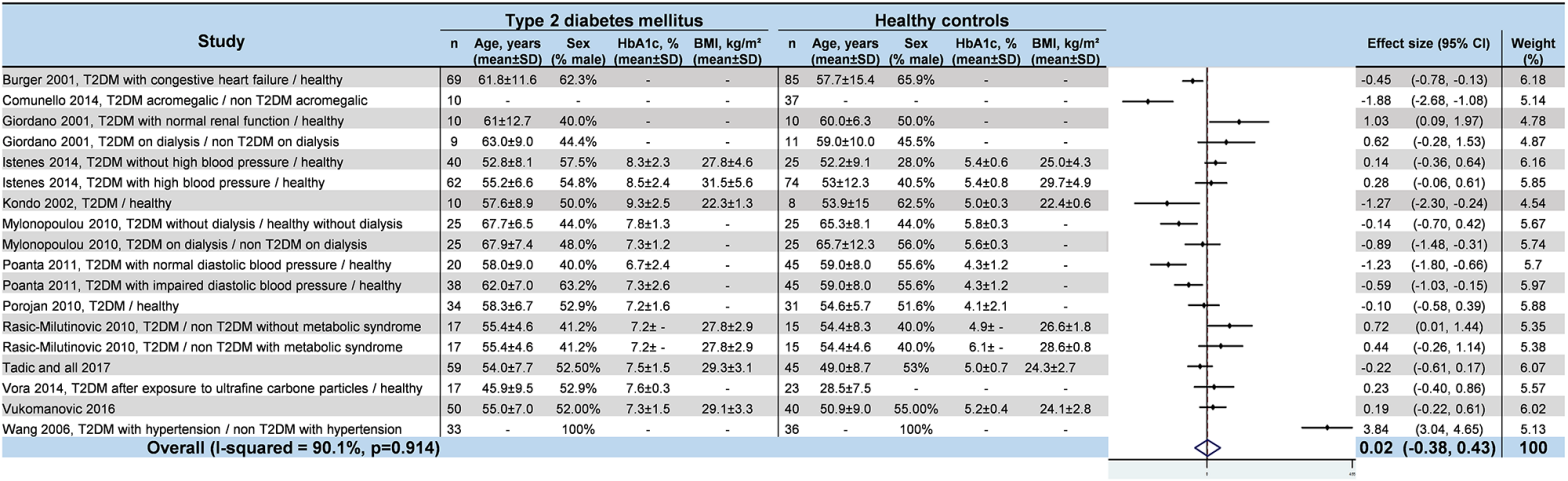
Meta–analysis of LF/HF of type 2 diabetes mellitus patients compared with controls. -: non reported data (missing SD were also non reported). 95% CI: 95% confident intervals; BMI: Body Mass Index; LF/HF: Low Frequency/High Frequency ratio; T2DM: Type 2 Diabetes Mellitus; SD: Standard Deviation.

We noted strong evidence that T2DM patients had significantly lower *RR intervals* (effect size = –0.61; 95%CI –1.21 to –0.01, P = 0.01; I^2^ = 91.6%) (Fig 2) [45,46,48,52,54,57], lower *SDNN* (effect size = –0.65; 95%CI –0.83 to –0.47; P < 0.001; I^2^ = 65.1%) (Fig 3) [42,45,47,48,50–53,55,57,58,60–66], lower *RMSSD* (effect size = –0.92; 95%CI –1.37 to –0.47); P < 0.001; I^2^ = 94.0%) (Fig 4) [45,47,48,50,52,53,56–58,60–66], lower *pNN50* (effect size = –0.46; 95%CI –0.84 to –0.09; P = 0.001; I^2^ = 85.5%) (Fig 5) [42,48,50–53,58,60,62,65,66], lower *total power* (effect size = –1.52; 95%CI –2.13 to –0.91; P < 0.001; I^2^ = 93.5%) (Fig 6) [45–48,50,52,54,65,66], lower *LF* (effect size = –1.08; 95%CI –1.46 to –0.69; p < 0.001; I^2^ = 91.3%) (Fig 7) [42,45,47–54,57,63,65,66], and lower *HF* (effect size = –0.79; 95%CI –1.09 to –0.50; P < 0.001; I^2^ = 85.6%) (Fig 8) [42,44,45,47–54,57,63,65,66]. *LF/HF* did not differ between group*s* (effect size = 0.02; 95%CI –0.38 to 0.43; P = 0.914; I^2^ = 90.1%) (Fig 9) [42,44,46–48,50–54,57,65,66]. Heterogeneity was significant (P < 0.001) for all meta–analyses. Funnel plots of meta–analyses analysing for potential publication bias are presented in the supplementary file. Meta–analyses were reperformed after the exclusion of studies that were not evenly distributed around the base of the funnel and showed similar results.

### Meta–regressions

**Fig 10:**
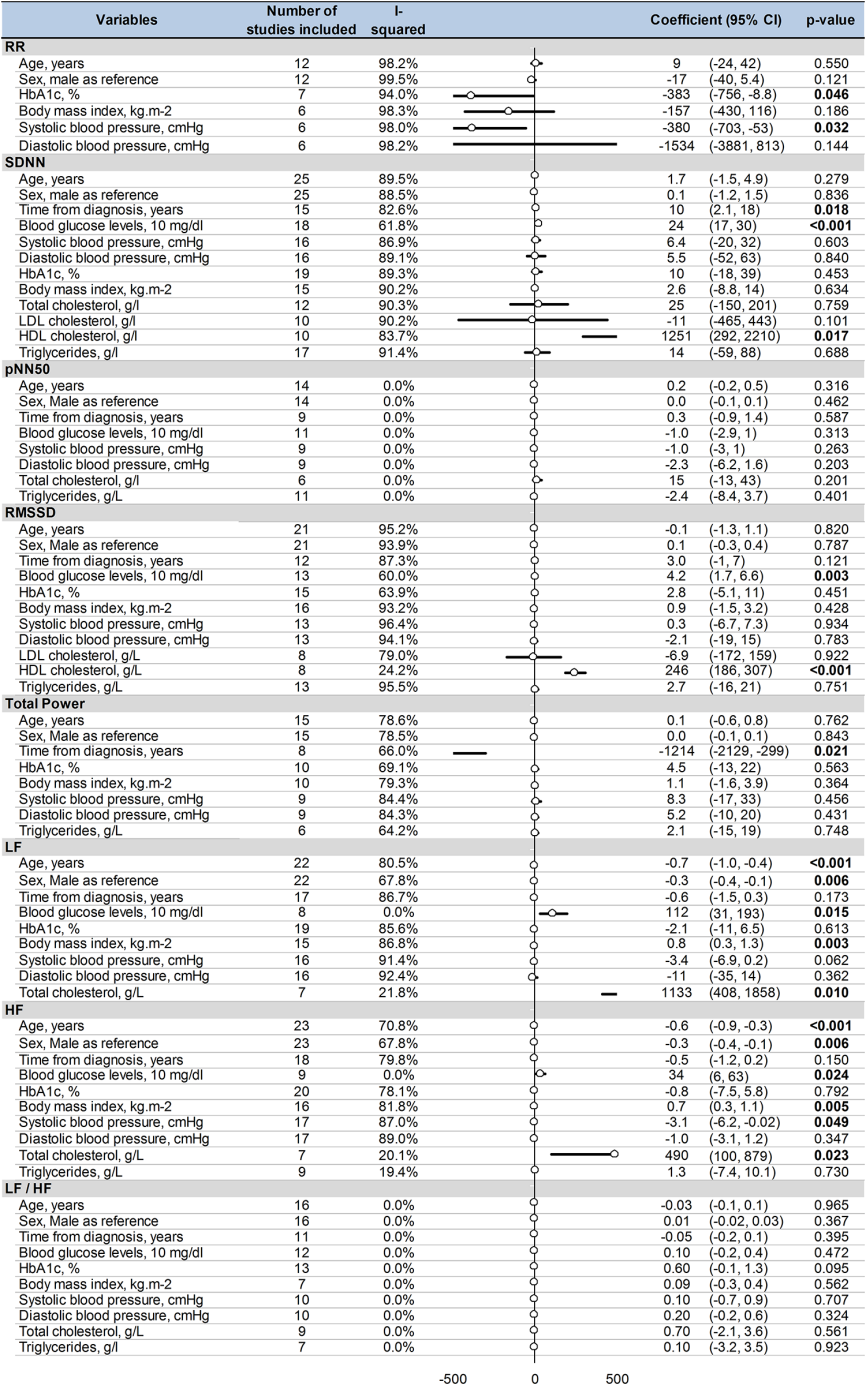
Meta–regression of factors influencing heart rate variability in type 2 diabetes mellitus. 95% CI: 95% confident intervals; RR: RR intervals; SDNN: Standard Deviation of RR intervals; pNN50: percentage of RR intervals with more than 50 ms variation; RMSSD: square root of mean squared differences of successive RR intervals; TP: Total Power; LF: Low Frequency; HF: High Frequency; LF/HF: Low Frequency/High Frequency ratio.

*Age* and *male* gender were associated with both a decrease in LF (coefficient = –0.7; 95%CI –1 to –0.4, P < 0.001; and coefficient = –0.3; 95%CI –0.4 to –0.1, P = 0.006, respectively) and a decrease in HF (coefficient = –0.6; 95%CI –0.9 to –0.3, P < 0.001; and coefficient = –0.3; 95%CI –0.4 to –0.1, P = 0.006). Similarly, both *blood glucose levels* and *total cholesterol* were associated with both an increase in LF (coefficient = 112; 95%CI 31–193, P = 0.015; coefficient = 1133; 95%CI 408–1858, P = 0.01; respectively) and in HF (coefficient = 34; 95%CI 6–63, P = 0.024; coefficient = 490; 95%CI 100–879, P = 0.023). *Blood glucose* levels were also significantly associated with an increase in RMSSD (coefficient = 4.2; 95%CI 1.7–6.6, P = 0.003) and with an increase in SDNN (coefficient = 24; 95%CI 17–30, P < 0.001). Higher levels of *HbA1c* were associated with shorter RR intervals (coefficient = –383; 95%CI –756 to –8.8, P = 0.046). *HDL cholesterol* levels were associated with both an increase in SDNN (coefficient = 1251; 95%CI 292–2210, P = 0.017) and RMSSD (coefficient = 246; 95%CI 186–307, P < 0.001). *Body mass index* was associated with both an increase in LF (coefficient = 0.8; 95%CI 0.3–1.3, P = 0.003) and in HF (coefficient = 0.7; 95%CI 0.3–1.1, P = 0.005). An increase in *systolic blood pressure* was linked with shorter RR intervals (coefficient = –380; 95%CI –703 to –53, P = 0.032) and a decrease in HF (coefficient = –3.1; 95%CI –6.2 to –0.02, P = 0.049). *Time from diagnosis* of T2DM was linked with a higher level of SDNN (coefficient = 10; 95%CI 2.1–18, P = 0.018) and a lower level of total power (coefficient = –1214; 95%CI –2129 to –299, P = 0.021) (Fig 10).

## Discussion

The main findings were that T2DM patients exhibited a strong decrease in HRV. Both sympathetic and parasympathetic activity were decreased compared with non–T2DM patients, which can be explained by the deleterious metabolic effects of blood glucose levels on HRV. Dyslipdaemia and high blood pressure are linked with a decreased HRV in T2DM. Finally, age and the male gender were associated with both a decrease in sympathetic and parasympathetic activity.

### An alteration of both sympathetic and parasympathetic activity

Adaptation to stress is characterized by an increase in sympathetic activity and a decrease in parasympathetic activity, inducing a state of alertness [17]. Interestingly, common diseases such as rheumatoid arthritis [67], depression [68], schizophrenia [69], multiple sclerosis [70], active ulcerative colitis [71], obesity and metabolic syndrome [72,73], myocardial infarctions [74], high blood pressure [75], smoking [76], and cancer [77], are associated with a decrease in parasympathetic activity and an activation of sympathetic activity. However, we demonstrated that T2DM patients had a decrease in both parasympathetic and sympathetic activity. An explanation could be that T2DM is a metabolic disease responsible for a cardiac autonomic neuropathy that affects both sympathetic and parasympathetic fibers. With the exception of the LF/HF ratio, which did not differ in our meta–analysis due to comparable changes in the LF and HF components, T2DM has a negative influence on almost all HRV parameters, reflecting the fact that diabetes leads to a cardiac autonomic dysfunction.

### Deleterious effects of altered glucose metabolism

The significant relationship between altered glucose metabolism and HRV may explain the deleterious general metabolic effects on both parasympathetic and sympathetic activity. Interestingly, blood glucose levels were associated with both an increase in LF (sympathetic) and HF (parasympathetic), as well as SDNN (sympathetic) and RMSSD (parasympathetic), which may appear contradictory. In healthy individuals, parasympathetic activity is triggered by an increase in blood glucose levels through insulin responses [78]. Unfortunately, insulin levels were not reported in most studies included in our meta–analysis, which precluded further analysis. Another explanation of this relation that could appear contradictory is that blood glucose levels are not a good marker to evaluate diabetes control compared with HbA1c: blood glucose levels reflect an instantaneous level whereas HbA1c reflect diabetes control over the previous months. In almost all included studies, patients had blood sampling during hospitalisation, after a steady state and in a controlled environment for diet with a close monitoring of capillary blood glucose, which may explain our contradictory results. Another convincing point is that the relationships between HbA1c and HRV is logic. We demonstrated that higher levels of HbA1c were associated with shorter RR intervals, which were associated with an increased risk of ventricular arrhythmias [79]. There was also a tendency for higher LF/HF ratio (i.e. decreased HRV) with higher levels of HbA1c. Furthermore, time from diagnosis of T2DM was linked with a higher level of SDNN. Despite we demonstrated a decreased SDNN in T2DM patients, the metaregression is not contradictory but may simply highlights the fact that cardiac parasympathetic activity in T2DM is affected before sympathetic activity [80]. Time from diagnosis of T2DM was also linked with a lower level of total power, but not with LF and HF. Thus we can hypothesize that very low frequencies (VLF) could be decreased in T2DM. No studies included in our meta–analysis reported VLF. Although less commonly used, VLF are recognised as the most powerful independent predictors of mortality in patients with heart failure or in patients with chronic haemodialysis [81]. Despite few studies assessing VLF in diabetes, interesting relationships were reported between VLF and sleep apnea in diabetics [82]. The potential significance of VLF in diabetes should be studied further.

### Other variables linked with HRV in T2DM

Similarly, total cholesterol was associated with both an increase in LF and HF, and HDL was associated with an increase in SDNN and RMSSD. To our knowledge, there is no data on hypercholesterolemia and HRV in the literature. Interestingly, some studies showed that a decrease in LDL by statin therapy could improve HRV parameters [83,84]. We demonstrated that an increase in systolic blood pressure was linked with shorter RR intervals and a decrease in HF. Despite no study previously assessing this relationship in diabetes, conflicting results were reported in the general population, with either high blood pressure associated with an increase in all spectral parameters [85], or a decrease in HRV [86]. It has also been suggested that the decrease in autonomic nervous function precedes the development of clinical hypertension [87]. Moreover, we found a significant relationship between BMI and HRV. Such relationships have been either found [88,89] or not [90,91] in the literature. However, the severity of obesity–related diseases is not directly linked to the accumulation of total body fat but rather to its distribution, and particularly to visceral localization [92]. HRV parameters have been previously correlated with sagittal abdominal diameter, anterior forearm skinfold thickness [93] and waist–hip ratio [90]. HRV parameters can also be improved after weight loss [94]. Finally, in line with the literature, we demonstrated a decrease in both LF and HF with age [95] and the male gender [96]. However, age and gender have a minor role on HRV parameters compared with the variables linked to T2DM.

### Limitations

All meta–analyses have limitations [97]. Meta–analyses inherit the limitations of the individual studies of which they are composed and are subjected to a bias of selection of included studies. However, the use of broader keywords in the search strategy limits the number of missing studies. Despite our rigorous criteria for including studies in our meta–analysis, their quality varied. Indeed, most of the studies included were cross–sectional, with different measurement conditions for HRV parameters associated with a high inter and intra–individual variability. We demonstrated that all parameters measuring HRV were significantly decreased in T2DM compared with controls. Though there were similarities between the participants’ inclusion criteria, they were not identical. Moreover, the health status of controls was not detailed in all studies, which could have influenced HRV parameters. This may have also minimized the differences in HRV between T2DM patients and controls. In addition, some studies were monocentric, limiting the generalizability of our results. However, included studies were homogeneous according to funnel plots, and the populations investigated in the meta–analysis appeared to be equally distributed around the world. Similarly, the final number of patients included in the metaanalysis was not very high and may precluded generalizability, however the mean age could be considered as quite representative. Despite missing data, our meta–regressions demonstrated significant and interesting relationships, particularly between HRV parameters and variables linked with T2DM. Variables retrieved from declarative data in each study included are also a putative bias.

## Conclusion

We reported strong evidence for an overall decrease in HRV in T2DM patients. Both sympathetic and parasympathetic activity were decreased, which can be explained by the deleterious effects of altered glucose metabolism on HRV. The benefits of an HRV evaluation in assessing and monitoring the severity of T2DM should be further studied, given its potential as a non–invasive, reliable and pain–free measurement.

## Supporting information

S1 Fig Quality score of included articles. (TIF)

S1 Appendix Example of search strategy on PubMed database. (PDF)

S2 Appendix PRISMA checklist. (DOCX)
