# Supplementary figures and images for: Heart rate variability in type 2 diabetes mellitus: A systematic review and meta–analysis
Source: PLoS One. 2018 Apr 2;13(4):e0195166. doi: 10.1371/journal.pone.0195166 (PMC5880391; doi:10.1371/journal.pone.0195166)

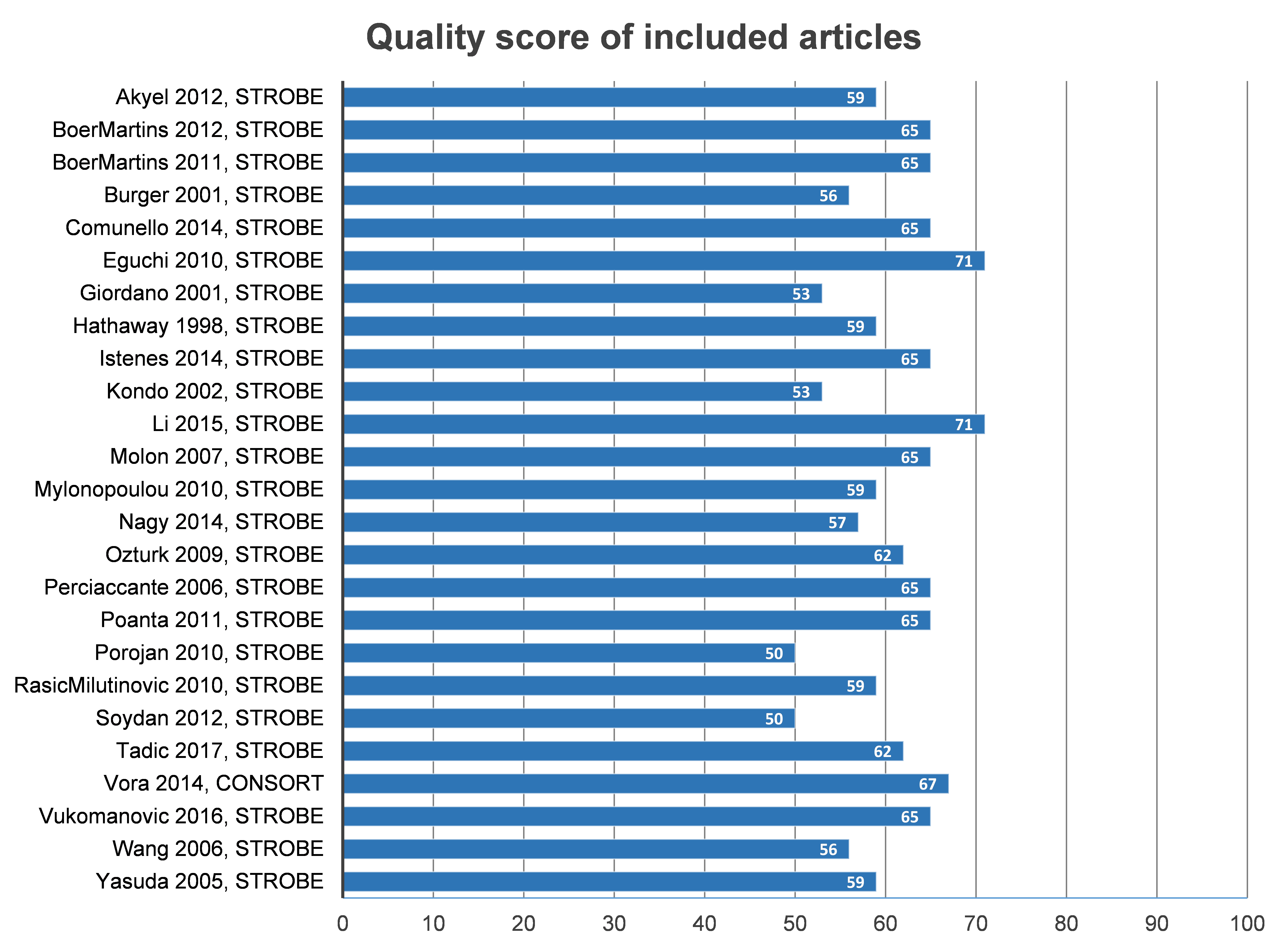

Supplement: S1 Fig — (TIF) [file pone.0195166.s001.tif]
