# Supplementary material for: Heart rate variability in type 2 diabetes mellitus: A systematic review and meta–analysis
Source: PLoS One. 2018 Apr 2;13(4):e0195166. doi: 10.1371/journal.pone.0195166 (PMC5880391; doi:10.1371/journal.pone.0195166)
Supplement: S1 Appendix — (PDF) [file pone.0195166.s002.pdf]

S1 Appendix. Example of search strategy on PubMed database.

1. Go to the Pubmed official page https://www.ncbi.nlm.nih.gov/pubmed/
2. In the search bar, write the following text: “diabetes AND (“heart rate variability” OR “HRV”)” https://www.ncbi.nlm.nih.gov/pubmed/?term=diabetes+AND+(%E2%80%9Cheart+rate+variability%E2%80%9D+OR+%E2%80%9CHRV%E2%80%9D)

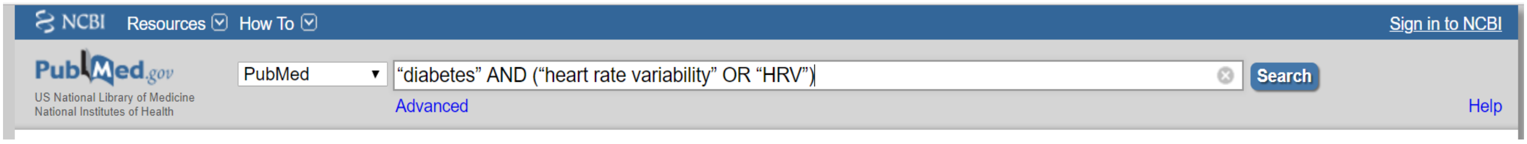

3. All results will appear
4. Then eligible articles need to be reviewed one by one
